# Multi-scale effects of soil and water conservation on runoff and sediment transport in a Chinese loess plateau basin

**DOI:** 10.1038/s41598-026-38546-z

**Published:** 2026-03-03

**Authors:** Xinhui Ding, Yuanhui Yu, Xiao Feng, Junjun Zhang, Xiaoying Liu

**Affiliations:** 1 College of Geography and Environment, Xianyang Normal University, Xianyang, 712000 China; 2 Yellow River Conservancy Technical University, Kaifeng, 475004 China; 3 International Research and Training Center on Erosion and Sedimentation, China Institute of Water Resources and Hydropower Research, Beijing, 100048 China; 4 Key Laboratory of Environmental Evolution and Ecological Restoration of Weibei Arid Plateau, Xianyang Normal University, Xianyang, China

**Keywords:** Multi-scale effects, Random forest, Soil and water conservation, Runoff and sediment reduction, Sanchuan river basin, Environmental sciences, Hydrology, Natural hazards

## Abstract

Quantifying the multi-scale effects of Soil and Water Conservation (SWC) measures is essential for managing soil erosion and water resources in the Yellow River Basin. This study evaluates the multi-scale (daily, monthly, annual) impacts of SWC on runoff and sediment reduction in the Sanchuan River Basin from 1960 to 2019 by integrating a Random Forest (RF) model with SHapley Additive exPlanations (SHAP) analysis. Key findings include: (1) Runoff reduction showed distinct seasonal variation, peaking at 53.8% in July, whereas sediment reduction remained consistently high (> 84%) year-round. (2) SHAP analysis quantitatively demonstrated that antecedent rainfall (R1d) exerted a stronger influence than current-day rainfall (R0d) on both runoff and sediment responses, underscoring the importance of cumulative hydrological conditions—a finding robust despite moderate model R² values (0.552 for runoff, 0.452 for sediment). (3) The analysis revealed two distinct benefit thresholds: significant runoff reduction emerged during 2001–2003 when terrace coverage reached 4.74 × 10⁴ hm², while peak sediment reduction occurred in 2013–2015 when forest area attained 18.94 × 10⁴ hm². These thresholds, derived from polynomial trend analysis and validated by Pettitt change-point detection, mark periods when cumulative SWC implementation is associated with significant hydrological benefits. The study provides a mechanistic, data-driven framework for understanding SWC effects and offers scale-specific, quantitative targets for adaptive watershed management in erosion-prone regions.

## Introduction

Since the 1960s, extensive soil and water conservation efforts have been implemented in the middle reaches of the Yellow River, achieving remarkable progress in management^1–4^. However, the multi-scale dynamics, key drivers, and critical thresholds of this effectiveness remain poorly quantified. Assessing the effectiveness of these measures is therefore paramount for guiding future management and ensuring the sustainability of water and soil resources^5,6^. Soil erosion, influenced by both climatic factors and human activities, alters watershed characteristics and consequently affects runoff and sediment discharge^7–10^. The implementation of SWC measures inevitably modifies these hydrological responses^11,12^. Crucially, the effectiveness of SWC measures is not static but exhibits complex temporal dynamics and may reach saturation points or thresholds^13,14^. Therefore, a comprehensive understanding of the multi-scale regulation of runoff and sediment by SWC measures—spanning from event-based daily responses to decadal trends—is essential.

To evaluate SWC effectiveness, two main approaches have been widely used: hydrological models (e.g., process-based models like SWAT) and empirical water conservation methods. Early studies often employed statistical methods to attribute runoff and sediment changes to climate and human activities^15^. Subsequent research has made significant strides in correlating SWC implementation area with watershed-scale reduction benefits and quantifying gross effects^16–18^. More recently, studies have incorporated more complex drivers and longer time series^5,19^. However, these approaches have inherent limitations. While hydrological models provide a process-based framework for scenario analysis, they often struggle to accurately capture nonlinear rainfall-sediment relationships. Conversely, empirical water conservation methods frequently rely on generalized reduction quotas that may not reflect local conditions^20^. Consequently, three critical knowledge gaps persist: (1) a lack of integrated analysis simultaneously revealing the distinct seasonal and long-term patterns of both runoff and sediment reduction benefits; (2) poorly quantified relative importance and interactive effects of immediate versus antecedent hydrological drivers across time scales; and (3) undefined critical implementation thresholds—in terms of both cumulative area and time—limiting precise management planning.

To address these gaps, this study introduces an integrated framework combining long-term hydrological analysis with interpretable machine learning. We employ a Random Forest (RF) model coupled with SHAP (SHapley Additive exPlanations) analysis to dissect the multi-scale effects of SWC in the Sanchuan River Basin from 1960 to 2019. The specific objectives are: (1) to quantify the daily, monthly, and annual-scale benefits of key SWC measures on runoff and sediment reduction; (2) to identify the dominant drivers, particularly the role of antecedent rainfall, and compare their influence on runoff versus sediment processes; and (3) to determine the critical threshold periods and associated implementation areas beyond which SWC measures yield substantial and sustained hydrological benefits. The findings aim to provide a mechanistic, evidence-based foundation for designing adaptive and scale-specific SWC strategies in the Loess Plateau and similar erosion-prone regions.

## Study area and methods

### Study area

**Fig. 1:**
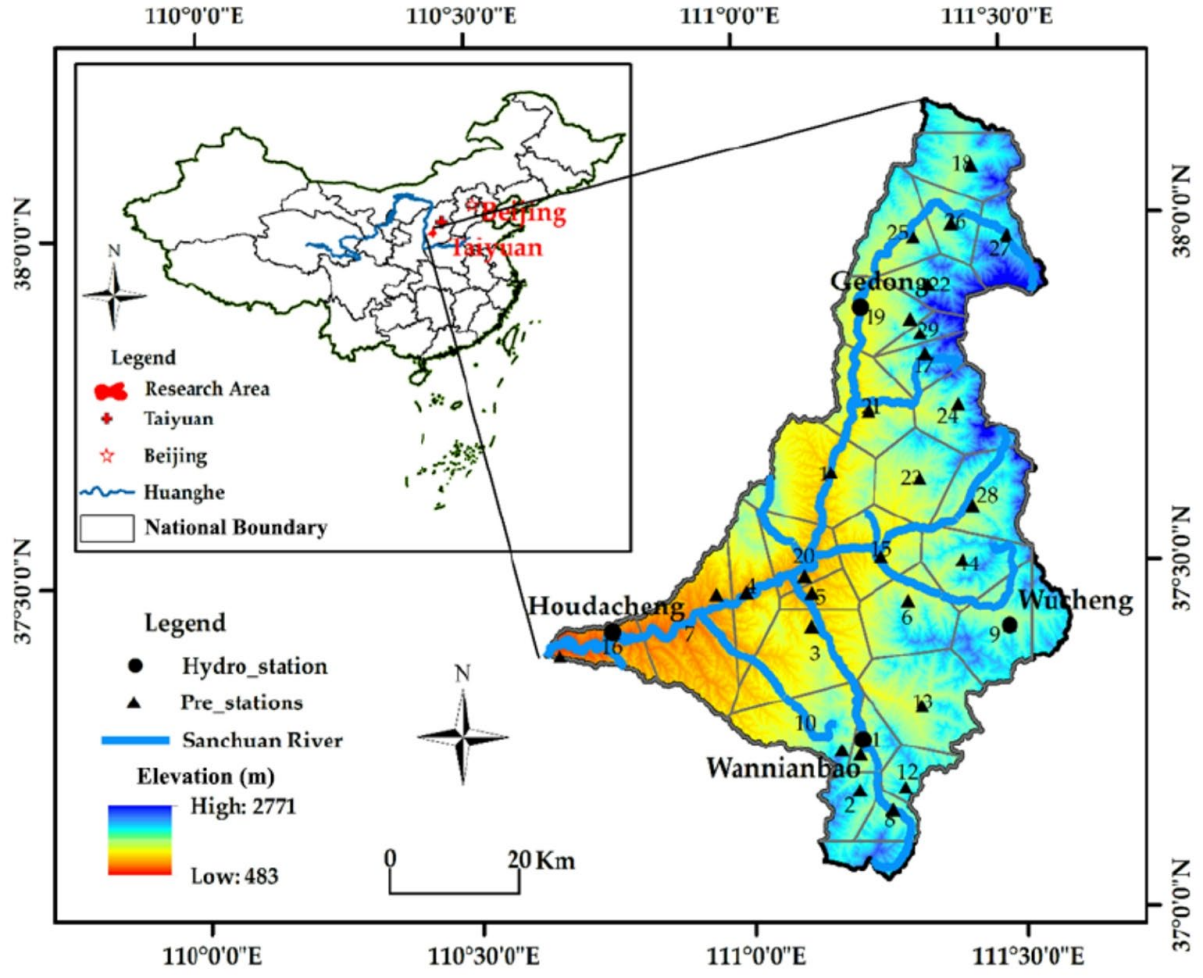
Locations of research site and hydrological stations in the Sanchuan River Basin.

The Sanchuan River Basin (35°52′–37°45′ N, 110°46′–111°28′ E) is located in the middle Yellow River reach and encompasses the Lüliang Mountain region of Shanxi Province, China (Fig. 1). The drainage network is formed by three primary tributaries—Beichuan, Dongchuan and Nanchuan rivers—that converge to create the Sanchuan River. The system drains four counties (Zhongyang, Liulin, Lishi and Fangshan), extends 176.4 km in main-channel length and covers 4,161 km². Characterized by a continental monsoon climate in the northern semi-arid zone, the basin has a mean annual temperature of 9.1 °C and a mean annual precipitation of 486 mm, of which > 70% falls between June and September. Precipitation is predominantly delivered by high-intensity rainfall events. For the purpose of this study, daily rainfall ≥ 50 mm is utilized as a key threshold for identifying significant erosive events, while rainstorms (≥ 100 mm) account for a substantial portion of the annual suspended-sediment yield. A hydrometric network consisting of four stream-gauging stations and 29 weighing-recording rain gauges provides continuous records of stage, discharge and precipitation at 15-min intervals; data are archived by the Shanxi Hydrological and Water Resources Survey Bureau.

### Data sources and processing

#### Hydrological and meteorological data

Long-term daily hydrological and meteorological data from January 1, 1960, to December 31, 2019, were utilized in this study. Daily records of runoff (m³/s) and sediment concentration (kg/s) were sourced from the Yellow River Hydrological Yearbook, with measurements recorded at the Houdacheng Hydrological Station, the basin’s controlling outlet. Precipitation data were collected from 29 observation stations distributed across the basin. To ensure data continuity and geographical representativeness, areal rainfall for the entire basin was calculated using the Thiessen polygon method, and spatial interpolation was performed using ArcGIS 10.8 to generate a continuous precipitation dataset.

#### Soil and water conservation (SWC) measures data

Data on the implementation of four key SWC measures—terracing, afforestation, grass planting, and check dam construction—were compiled from literature searches on the CNKI (China National Knowledge Infrastructure) platform. Gaps in the annual data series were filled using the Lagrange interpolation method to ensure a continuous timeline. Based on the historical implementation intensity of SWC measures and documented changes in runoff and sediment, the study period was divided into four distinct phases: Initial Treatment Period (1960–1979), Concentrated Treatment Period (1980–1996), Stable Period (1997–2009), and Effective Period (2010–2019).

**Fig. 2:**
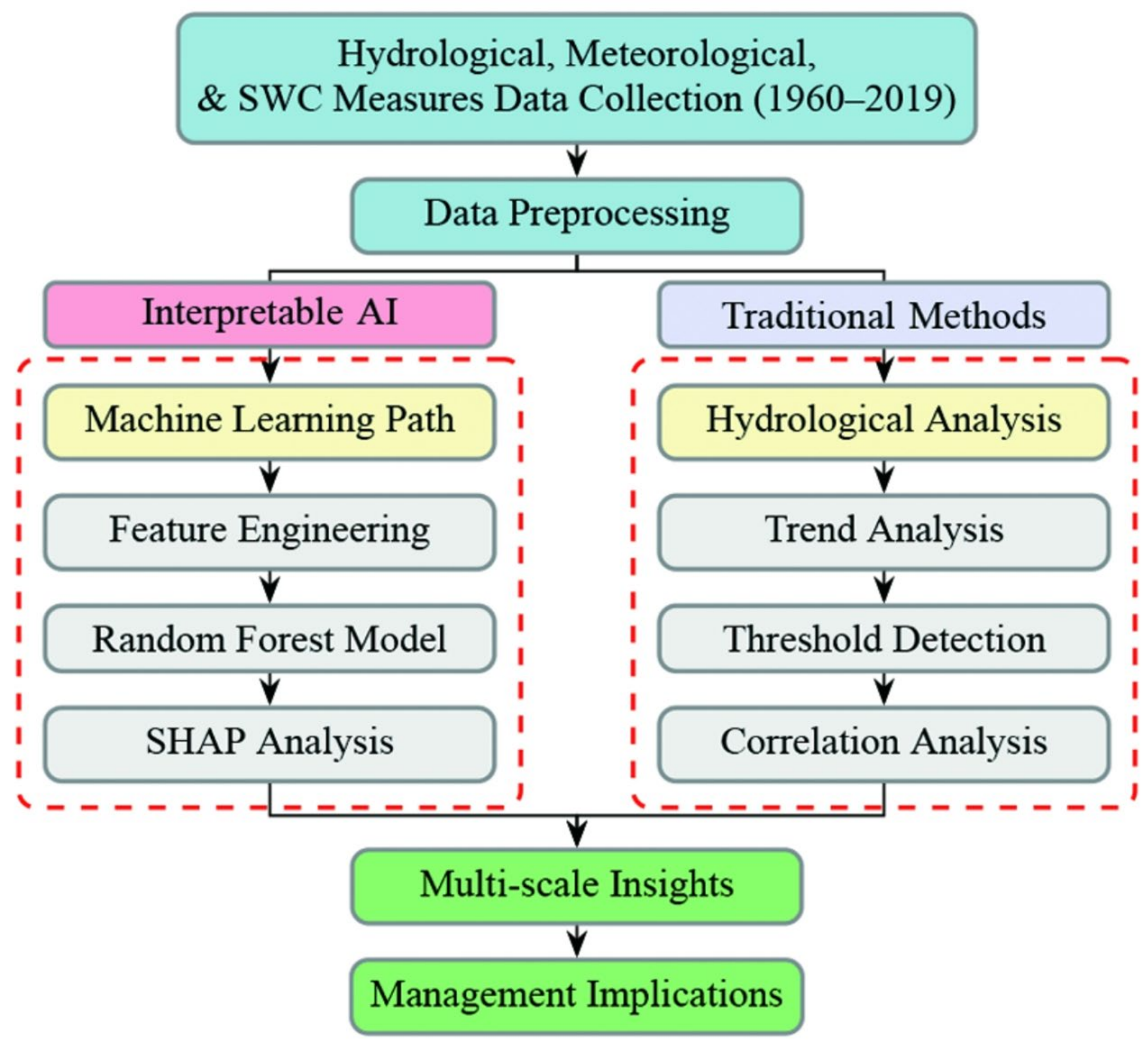
Methodological framework for multi-scale SWC assessment.

### Methodological approach

The analytical framework of this study (Fig. 2) integrates multiple approaches to assess the multi-scale effects of soil and water conservation measures. The methodology comprises three main components: quantification of SWC benefits, multi-scale feature engineering with machine learning modeling, and threshold analysis of conservation measures.

#### Quantification of SWC benefits

To quantify the hydrological benefits of SWC measures over the 60-year period, a baseline comparison approach was adopted. The 1960s was selected as the reference period, as it represents a phase prior to the large-scale implementation of SWC measures in the basin, thus approximating a near-natural hydrological baseline state.

For each daily observation, the runoff reduction benefit (*R*_*benefit*​_) and sediment reduction benefit (*S*_*benefit*​_) were calculated as the relative deviation from this baseline :

$$\:{\mathrm{R}}_{\mathrm{b}\mathrm{e}\mathrm{n}\mathrm{e}\mathrm{f}\mathrm{i}\mathrm{t}}=\frac{\mathrm{B}\mathrm{a}\mathrm{s}\mathrm{e}\_\mathrm{R}\:-\:\mathrm{R}\_\mathrm{o}\mathrm{b}\mathrm{s}}{\mathrm{B}\mathrm{a}\mathrm{s}\mathrm{e}\_\mathrm{R}}\times\:100\%$$

$$\:{\mathrm{S}}_{\mathrm{b}\mathrm{e}\mathrm{n}\mathrm{e}\mathrm{f}\mathrm{i}\mathrm{t}\:}=\frac{\mathrm{B}\mathrm{a}\mathrm{s}\mathrm{e}\_\mathrm{S}\:-\:\mathrm{S}\_\mathrm{o}\mathrm{b}\mathrm{s}}{\mathrm{B}\mathrm{a}\mathrm{s}\mathrm{e}\_\mathrm{S}}\times\:\:100\%$$

where Base_R and Base_S represent the long-term average daily runoff and sediment concentration for each corresponding month, derived from the 1960s reference period. Specifically, for a given day in May, Base_R (or Base_S) is calculated as the mean of all daily observations in May across the years 1960–1969. This approach establishes a within-month daily climatological baseline that characterizes the near-natural hydrological conditions before intensive SWC implementation. R_obs and S_obs are the observed daily values.

To mitigate the influence of extreme outliers that could arise from rare meteorological events, measurement uncertainties, or the inherent variability in the baseline comparison method—and to prevent such outliers from disproportionately influencing the subsequent machine learning model—the calculated daily benefit values were constrained to the range of -100% to + 100%. This is a common preprocessing step to enhance model stability and generalizability.

Finally, monthly and annual average benefit values were computed from these processed daily series for use in trend and threshold analysis.

#### Multi-scale feature engineering and random forest modeling

To capture hydrological processes across multiple temporal scales and enhance model interpretability, comprehensive feature engineering was performed on the daily observational data. Three categories of predictive features were constructed based on their hydrological significance: Daily-scale features included daily rainfall (Rainfall, mm) and its 1-day, 2-day, and 3-day lagged values (Rainfall_lag1, Rainfall_lag2, Rainfall_lag3) to account for antecedent moisture conditions, which are critical for runoff generation and sediment transport processes. Seasonal features incorporated trigonometric transformations of month (sin_month, cos_month) to capture cyclical hydrological patterns and seasonal variations in rainfall-runoff relationships. Temporal features comprised raw month (month) and year (year) variables to track long-term trends and anthropogenic impacts on hydrological processes. In total, eight features were used for model development.

Separate Random Forest (RF) regression models were developed to predict daily runoff reduction benefit (R_benefit) and sediment reduction benefit (S_benefit). The RF algorithm was selected for its capability to handle nonlinear relationships, resist overfitting, and provide feature importance measures. The modeling was based on 18,590 valid daily records after quality control. This dataset was partitioned into a training set (14,872 samples, 80%) and an independent test set (3,718 samples, 20%), with stratification by month to preserve seasonal representation in both sets. Model parameters were optimized through grid search with 5-fold cross-validation. The final RF models were parameterized with 500 trees to ensure stability, a maximum depth of 15 to balance model complexity and generalization, a minimum of 5 samples for node splitting, 2 samples per leaf, and the square root of the total number of features considered for each split. A fixed random state of 42 was used to ensure reproducibility. It is important to note that the areas of implemented SWC measures (e.g., terraces, forests) were not included as predictive features in these daily-scale models. Their impact is analyzed separately in the annual-scale threshold analysis (Sect. "Threshold analysis of SWC measures"). To enhance model interpretability and identify key drivers, SHAP (SHapley Additive exPlanations) analysis was employed. SHAP values quantify the contribution of each feature to individual predictions, providing physically interpretable insights into the temporal dynamics of rainfall impacts on basin responses.

#### Threshold analysis of SWC measures

In the context of the Loess Plateau, where large-scale and sustained SWC implementation has been the dominant anthropogenic driver of hydrological change since the 1960s, the systematic deviations from the baseline are interpreted as reflecting the cumulative effect of SWC measures, while acknowledging the potential modulating role of other factors.

To analyze the long-term trends of runoff and sediment and identify critical thresholds of soil and water conservation effectiveness, a comprehensive trend analysis was conducted. After multiple comparative tests with different smoothing parameters, a 3-year moving average was selected to reduce data dispersion while preserving interannual variability, as it optimally balanced noise reduction and trend preservation.

For curve fitting, we systematically evaluated polynomial orders from 2nd to 7th (Table 1). The 5th-order polynomial was selected as optimal based on: (1) lowest AIC values for both runoff (-62.10) and sediment (29.13); (2) substantially higher R² than lower-order models, with the most significant improvement from 4th to 5th order; and (3) avoidance of diminishing returns evident in 6th-7th orders (minimal R² gain but higher AIC).

**Table 1:** Polynomial order evaluation for runoff and sediment sequences.

Order	Runoff-*R*^2^	Runoff-AIC	Runoff-RMSE	Sediment-*R*^2^	Sediment-AIC	Sediment-RMSE
2	0.516	-54.08	1.70	0.302	35.99	0.52
3	0.526	-53.28	0.90	0.311	37.23	1.04
4	0.553	-54.78	4.12	0.368	33.99	7.77
5	0.617	-62.10	8.44	0.436	29.13	14.81
6	0.617	-60.08	8.51	0.436	31.15	14.96
7	0.617	-58.05	8.58	0.436	33.16	15.10

The 5th-order model exhibits higher validation RMSE, reflecting the inherent trade-off between historical fitting and predictive accuracy. This trade-off is justified given our research objective: identifying historical trend inflections takes priority over prediction accuracy. The clear improvements in AIC and R² support the model’s capability to capture the nonlinear transitions relevant to SWC cumulative effects.

Threshold periods were identified through inflection point analysis of the 5th-order polynomial curves, enabling calculation of runoff and sediment variations at each temporal node and identification of distinct threshold periods across management phases.

## Results

### Phase-based analysis of long-term trends in rainfall, runoff, and sediment (1960–2019)

**Fig. 3:**
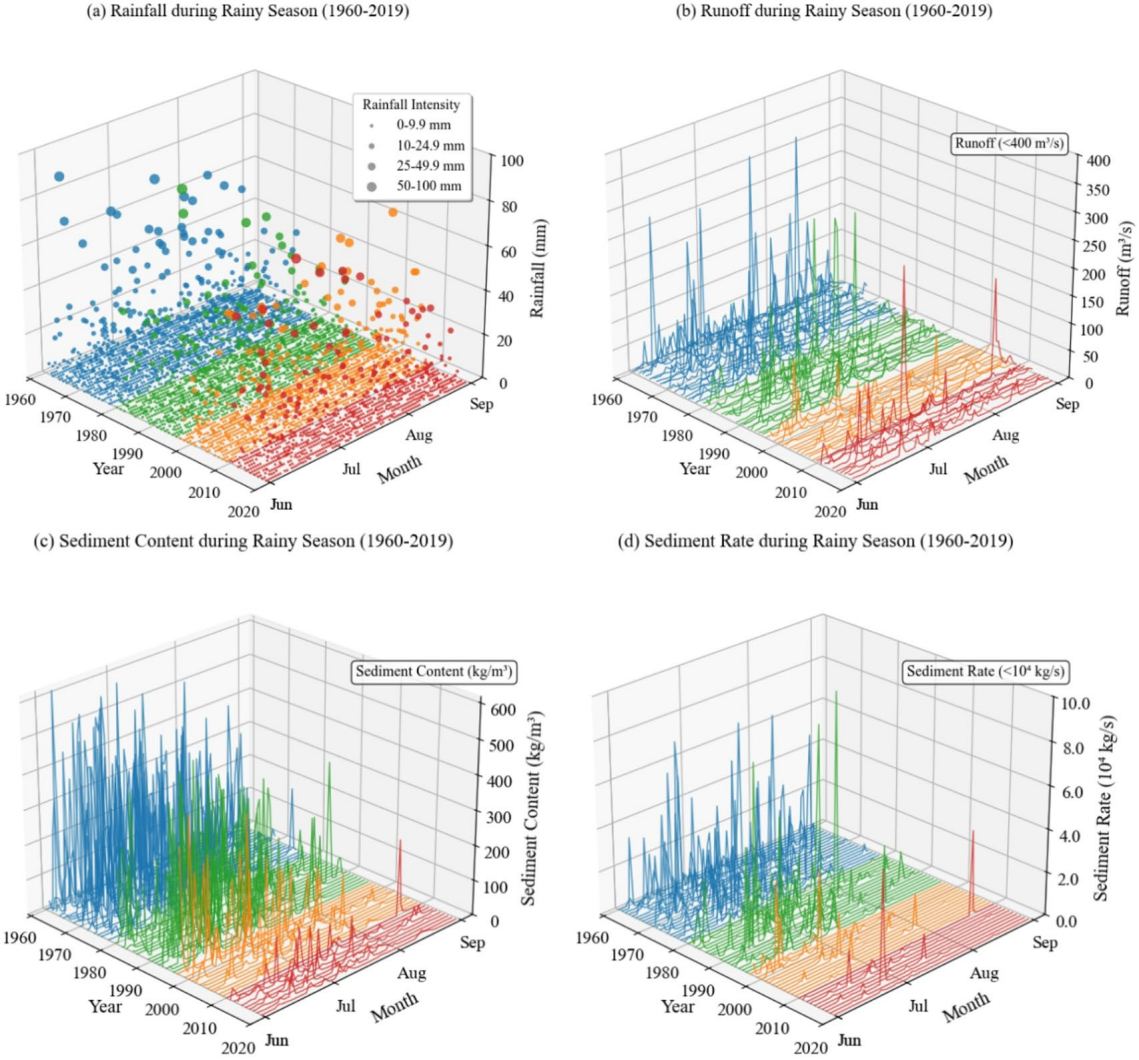
Long-term Trends in Rainfall, Runoff, Sediment Concentration, and Sediment Transport Rate during the Rainy Season (1960–2019). Note: Panels (**a**) and (**b**) show daily rainfall and runoff, respectively, with colors indicating four SWC management periods: Initial Treatment (1960–1979, blue), Concentrated Treatment (1980–1996, green), Stable Period (1997–2009, orange), and Effective Period (2010–2019, red). Panels (**c**) and (**d**) display sediment concentration and sediment transport rate, respectively. The stepwise decline in sediment-related variables across periods highlights the progressive effectiveness of SWC measures.

Figure 3 presents the long-term rainy-season (July to September) dynamics of rainfall, runoff, and sediment in the Sanchuan River Basin from 1960 to 2019. A unified color scheme is used to distinguish four distinct Soil and Water Conservation (SWC) management periods. Figure 3(a) illustrates the distribution of daily rainfall intensity during the rainy season. In the early period (1960–1979, blue), clusters of large-sized points—indicating consecutive days of high-intensity rainfall—are frequently observed. In contrast, during the recent period (2010–2019, red), large points appear predominantly in isolation. On a monthly scale, high-intensity events occur more frequently in July and August than in September. Notably, after the year 2000, such events have also been occasionally recorded in September. Figure 3(b) reveals a marked interdecadal decline in daily runoff magnitude. The early period (1960–1979, blue) shows frequent high runoff values, forming dense clusters near the top of the plot. Conversely, in the recent period (2010–2019, red), nearly all runoff values are concentrated at lower levels. Runoff is generally higher in July and August than in September, which aligns with the seasonal rainfall pattern. However, this seasonal contrast has diminished over time.

Figure 3(c) displays the three-dimensional distribution of sediment concentration. A high-sediment phase (1960–1979, blue) is characterized by substantial vertical fluctuations and frequent extreme values. This is followed by a transitional phase (1980–1996, green), during which high values are notably reduced in both frequency and magnitude. Subsequently, a low-sediment phase (1997–2019, orange/red) emerges, with concentrations stabilizing within a low range. Sediment concentration is typically highest in July, and inter-monthly differences have become less pronounced in recent decades. Figure 3(d) shows a pronounced interdecadal decline in sediment transport rate. In the early period (blue), values are widely distributed along the vertical axis, whereas in the later period (red), they are predominantly concentrated near the base of the graph. A comparison between Figs. 3(b) and 3(d) indicates that in the early period, even moderate runoff levels were associated with relatively high sediment transport rates. In the later period, however, sediment transport rates remain low despite the occurrence of runoff. In summary, the data indicate significant transformations in the basin’s hydrological and sediment regimes over the study period. The clustering of high-intensity rainfall events has decreased, while runoff and sediment transport have exhibited substantial declines in magnitude and intra-annual variability.

Further insight into extreme sediment dynamics is provided by the record of major sediment transport events (Table 2). All events with sediment transport rates exceeding 100,000 kg/s occurred between 1962 and 1988, predominantly during the early and intensive management phases. The most extreme event recorded was on July 18, 1966, with a sediment transport rate of 462,000 kg/s under 122.3 mm of rainfall, accompanied by a runoff of 827 m³/s and a sediment concentration of 558.65 kg/m³. Notably, a substantial sediment load (114,000 kg/s) was also transported on the preceding day (July 17, 1966) under much lighter rainfall (8.8 mm), highlighting the persistence of high sediment availability and transport capacity during consecutive storm events in the early, less-managed era. These events demonstrate that extreme sediment yields are often the product of high runoff combined with high sediment concentration, though exceptional sediment availability can lead to major transport events even under moderate runoff conditions, as seen on July 15, 1962.

**Table 2:** Selected Rainy-Season sediment events (1960–2019).

Date	Rainfall (mm)	Runoff (m³/s)	Sediment content (kg/m³)	Sediment rate(kg/s)
1962/7/15	35.5	600	531.6667	319,000
1963/7/6	89	283	452.2968	128,000
1964/9/7	66	332	400.6024	133,000
1966/7/17	8.8	229	497.8166	114,000
1966/7/18	122.3	827	558.6457	462,000
1967/8/22	47.6	563	426.2877	240,000
1969/8/9	18.8	353	441.9263	156,000
1977/7/6	83.3	336	479.1667	161,000
1977/8/5	64.2	236	432.2034	102,000
1977/8/6	59.6	410	382.9268	157,000
1988/8/6	46.7	299	377.9264	113,000

### Lag effects of rainfall on runoff and sediment transport

**Fig. 4:**
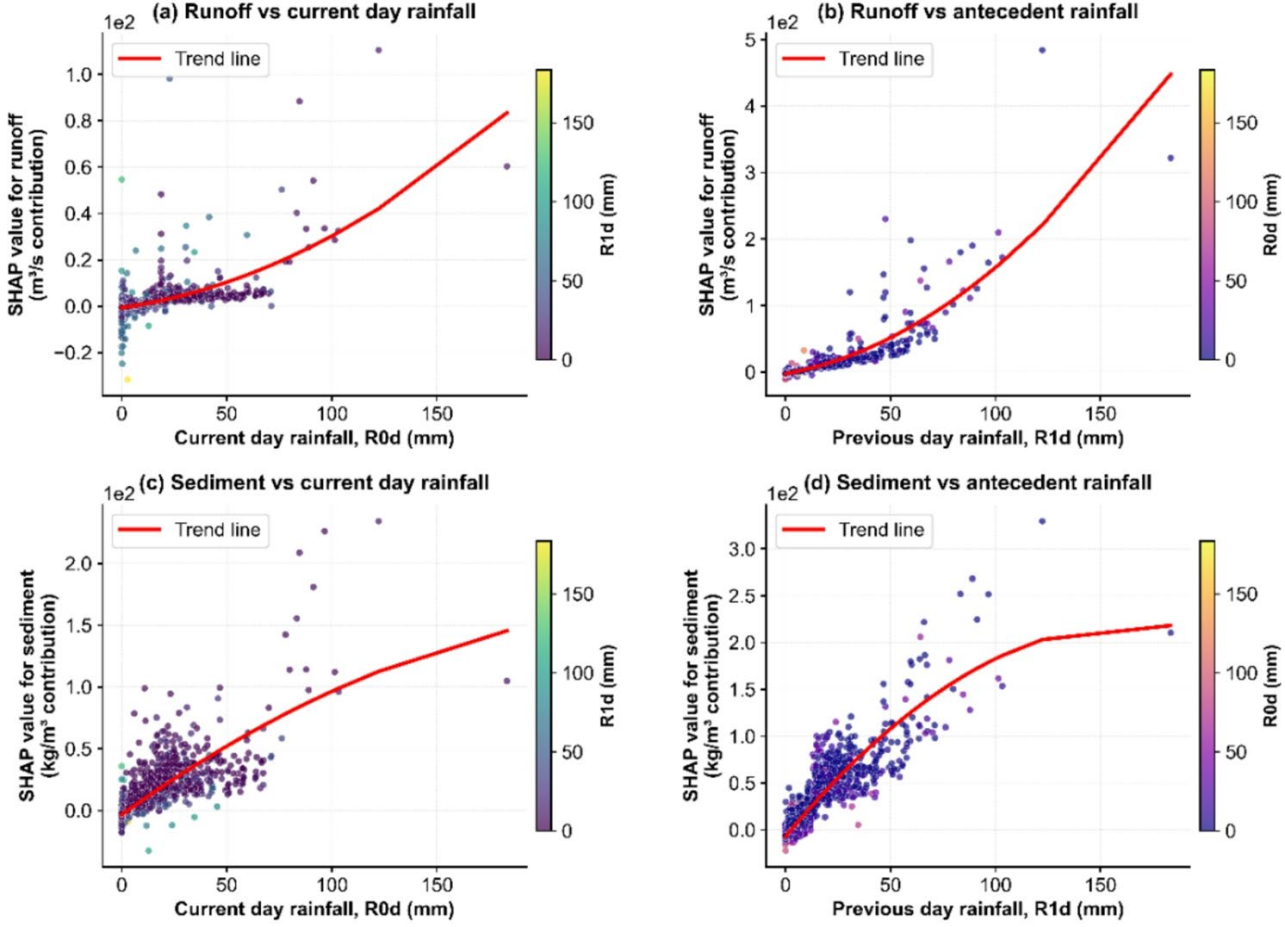
SHAP dependence plots illustrating lag effects of rainfall on runoff and sediment. Note: Each plot displays the relationship between a feature (e.g., 1-day lagged rainfall) and its SHAP value, which quantifies the feature’s contribution to the model output. Points are colored by the magnitude of another correlated feature (e.g., current rainfall) to reveal interaction effects.

Building on the observed temporal patterns, we employed SHAP analysis to quantify the driving factors. The SHAP dependence plots reveal distinct lagged effects of antecedent rainfall on runoff generation and sediment transport processes in the Sanchuan River Basin (Fig. 4). On the independent test set, the Random Forest models achieved a coefficient of determination (R²) of 0.552 for runoff, with a root mean square error (RMSE) of 26.92% and a mean absolute error (MAE) of 17.18%. For sediment, the model performance was characterized by an R² of 0.452, an RMSE of 30.10%, and an MAE of 11.63%. On the training set, the models achieved an R² of 0.704 and an RMSE of 21.71% for runoff, and an R² of 0.640 and an RMSE of 23.80% for sediment. These performance indicators confirm that the models capture a substantial, yet incomplete, portion of the variance characterizing these highly stochastic, daily-scale hydrological processes. The moderate R² values reflect the inherent challenge of fully explaining all variability; however, the associated error magnitudes remain within a practically informative range for such complex systems. Importantly, the subsequent SHAP analysis provides robust, physically interpretable insights into the dominant drivers and their temporal dynamics, which is the primary objective of this study. The analysis consistently identifies antecedent rainfall (R1d) as a factor with stronger explanatory power than current-day rainfall (R0d) for both runoff and sediment responses. This pattern is particularly pronounced for sediment concentration, indicating that sediment transport processes are more strongly influenced by preceding hydrological conditions than by immediate precipitation. This key finding derives from the stable relative importance patterns learned by the model, thereby offering valid mechanistic insight even in the absence of perfect predictive accuracy. Notably, the analysis demonstrates that antecedent rainfall (R1d) exhibits stronger explanatory power than current-day rainfall (R0d) for both hydrological responses. The correlation coefficients for sediment concentration are particularly striking, indicating that sediment transport processes are more strongly influenced by preceding rainfall conditions than immediate precipitation events.

The mean absolute SHAP values further quantify this lag dominance. For runoff generation, the influence of R1d (3.996 m³/s) is approximately four times greater than R0d (1.048 m³/s), suggesting that soil moisture conditions from previous rainfall play a crucial role in runoff formation. Similarly, for sediment transport, R1d’s contribution (12.019 kg/m³) nearly doubles that of R0d (6.108 kg/m³), highlighting the importance of antecedent basin conditions in sediment mobilization. These findings align with the observational data from extreme events. The July 1966 sequence, where substantial sediment transport occurred on consecutive days (114,000 t on July 17 and 462,000 t on July 18 under 122.3 mm rainfall), exemplifies the cumulative effect captured by the P1d dominance. The SHAP results provide a mechanistic explanation for why consecutive rainfall events during the early management period produced persistently high sediment yields, as the model identifies antecedent conditions as the primary driver.

The stronger lag effects for sediment transport compared to runoff generation suggest that sediment-related processes have longer memory effects in the basin system. This may be attributed to the time required for sediment detachment, transport capacity development, and the legacy effects of previous erosion events on sediment availability.

Furthermore, the reduction in runoff and sediment responses to light and moderate rainfall across management periods, as observed in Fig. 4, can be reinterpreted through the SHAP lens. The implementation of soil and water conservation measures likely altered the rainfall-runoff-sediment relationships by modifying how antecedent conditions influence current responses, particularly breaking the cumulative effects captured by the R1d dominance in the early management period.

### Temporal patterns of runoff and sediment change indicators

**Fig. 5:**
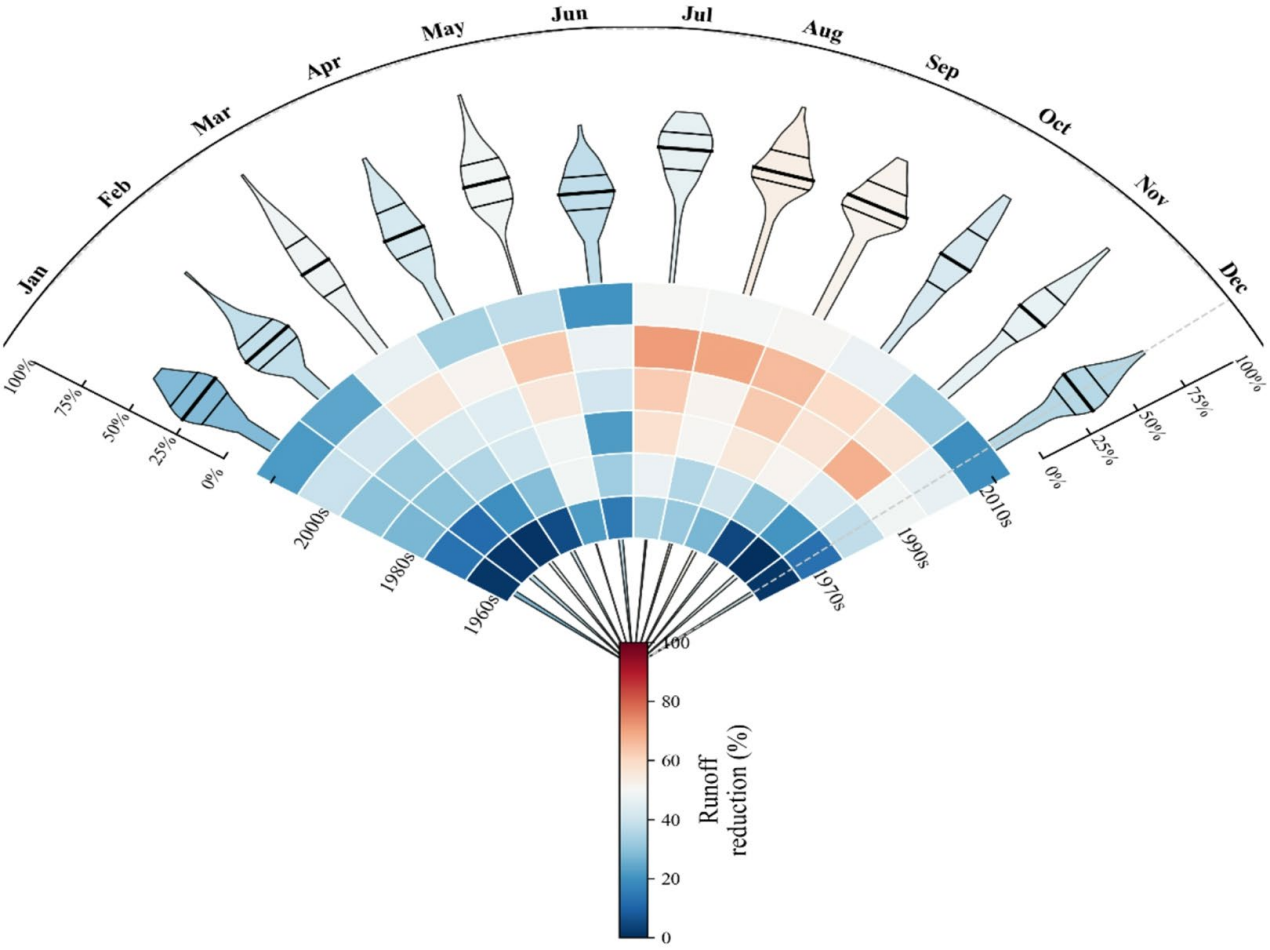
Runoff reduction benefits Over 1960–2019 s in the Sanchuan River Basin.

The identified dominance of antecedent conditions at the daily scale provides a mechanistic underpinning for the more stable, cumulative benefits observed at longer (monthly to annual) time scales. Based on the relative change indicators (R_benefit, S_benefit) calculated in Sect. "Quantification of SWC benefits", we analyzed their temporal patterns at monthly and decadal scales. The Random Forest machine learning model, combined with SHAP interpretability analysis, was employed to obtain these reduction indicators. The results indicate significant temporal variations in runoff reduction benefits. On a monthly basis, the most pronounced runoff reduction benefits were observed in May, July, August, and September, with the highest average value of 53.8% in July (Fig. 5). In contrast, the lowest benefits were recorded in November and December, with average values of 36.7% and 27.7%, respectively. This suggests that runoff reduction measures are particularly effective during the rainy season and flood season, especially in the summer months. In terms of decadal changes, runoff reduction benefits exhibited a clear trend of initial increase followed by a decline. The average benefit increased steadily from 12.0% in the 1960s to 55.5% in the 2000s, representing a net rise of 3.6 times. This upward trend reflects the continuous improvement and cumulative effects of soil and water conservation measures. However, a significant decrease was observed in the 2010s, with the average benefit dropping to 36.0%, a decline of 35% compared to the 2000s. This decline may be attributed to factors such as increased extreme rainfall events under climate change or changes in land use.

**Fig. 6:**
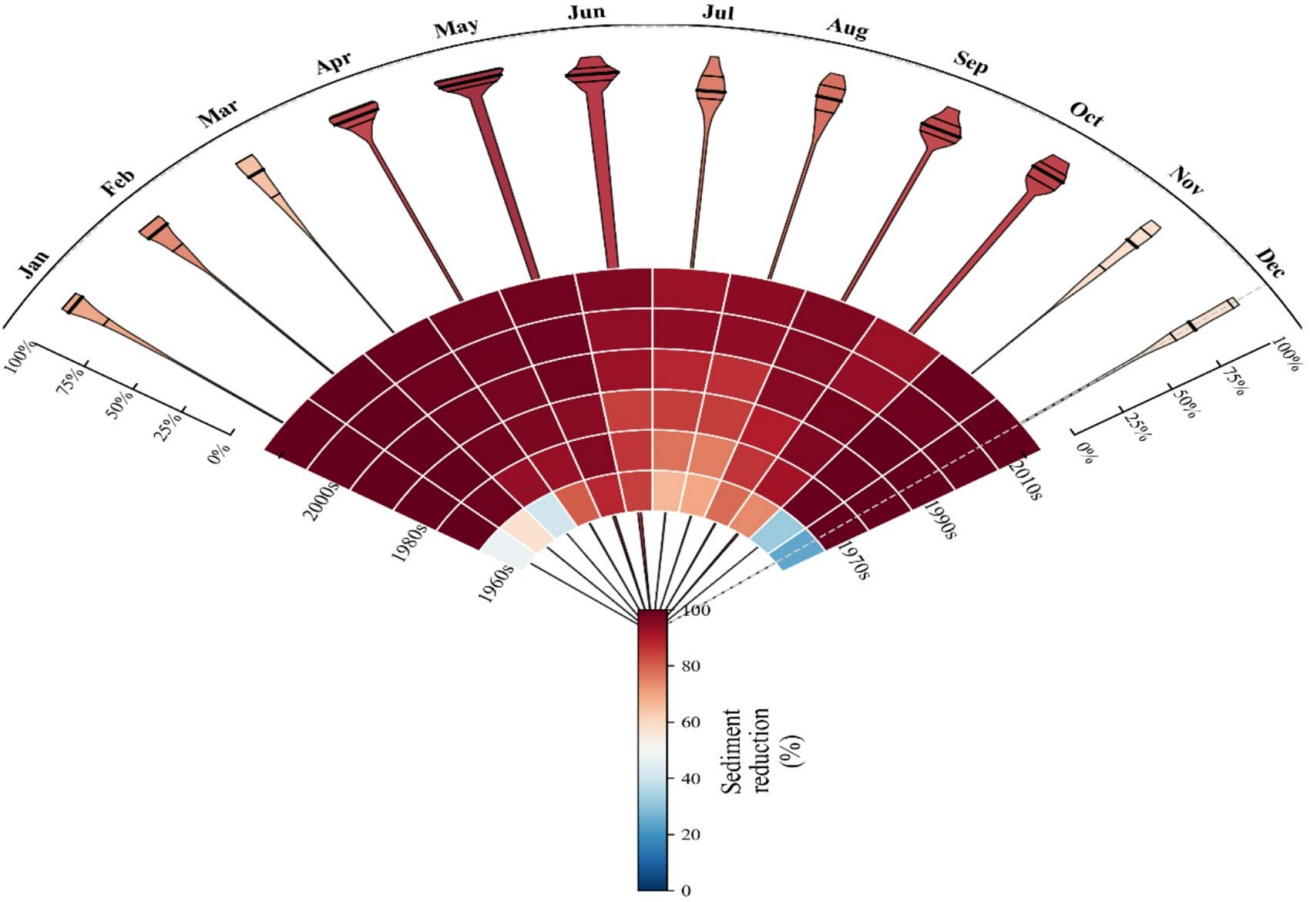
Sediment reduction benefits Over 1960–2019 s in the Sanchuan River Basin.

The temporal distribution characteristics of sediment reduction benefits differ from those of runoff reduction benefits (Fig. 6). On a monthly basis, sediment reduction benefits remained consistently high throughout the year, with the most significant benefits observed in April, May, and the winter months (January, February, and December), where the average benefit exceeded 90%. In contrast, the benefits in July and August were relatively lower, but still maintained at a high level above 84%. This indicates that soil conservation measures provide stable, year-round control of sediment. The decadal analysis of sediment reduction benefits revealed a continuous improvement trend. The average benefit increased significantly from 61.8% in the 1960s to 91.3% in the 1970s, and continued to optimize in subsequent decades, reaching the highest level of 97.3% in the 2010s. This sustained improvement reflects the long-term cumulative effects of soil conservation measures, such as afforestation, terraced field construction, and improved agricultural practices, particularly in controlling soil erosion.

The comprehensive analysis indicates that soil and water conservation measures have demonstrated excellent and stable performance in reducing sediment, with an average benefit of 89.6% and a maximum benefit reaching 100%. In contrast, the benefits in reducing runoff, although relatively lower (with an average of 37.2%), still play an important role in specific seasons and periods. The differences in the temporal distribution of the two types of benefits suggest that future soil and water conservation strategies need to be optimized and adjusted according to seasonal characteristics and long-term climate change trends. In particular, measures should be strengthened to address the challenge of the decline in runoff reduction benefits since the 2010s.

### Threshold analysis of soil and water conservation measures in the Sanchuan river basin

**Fig. 7:**
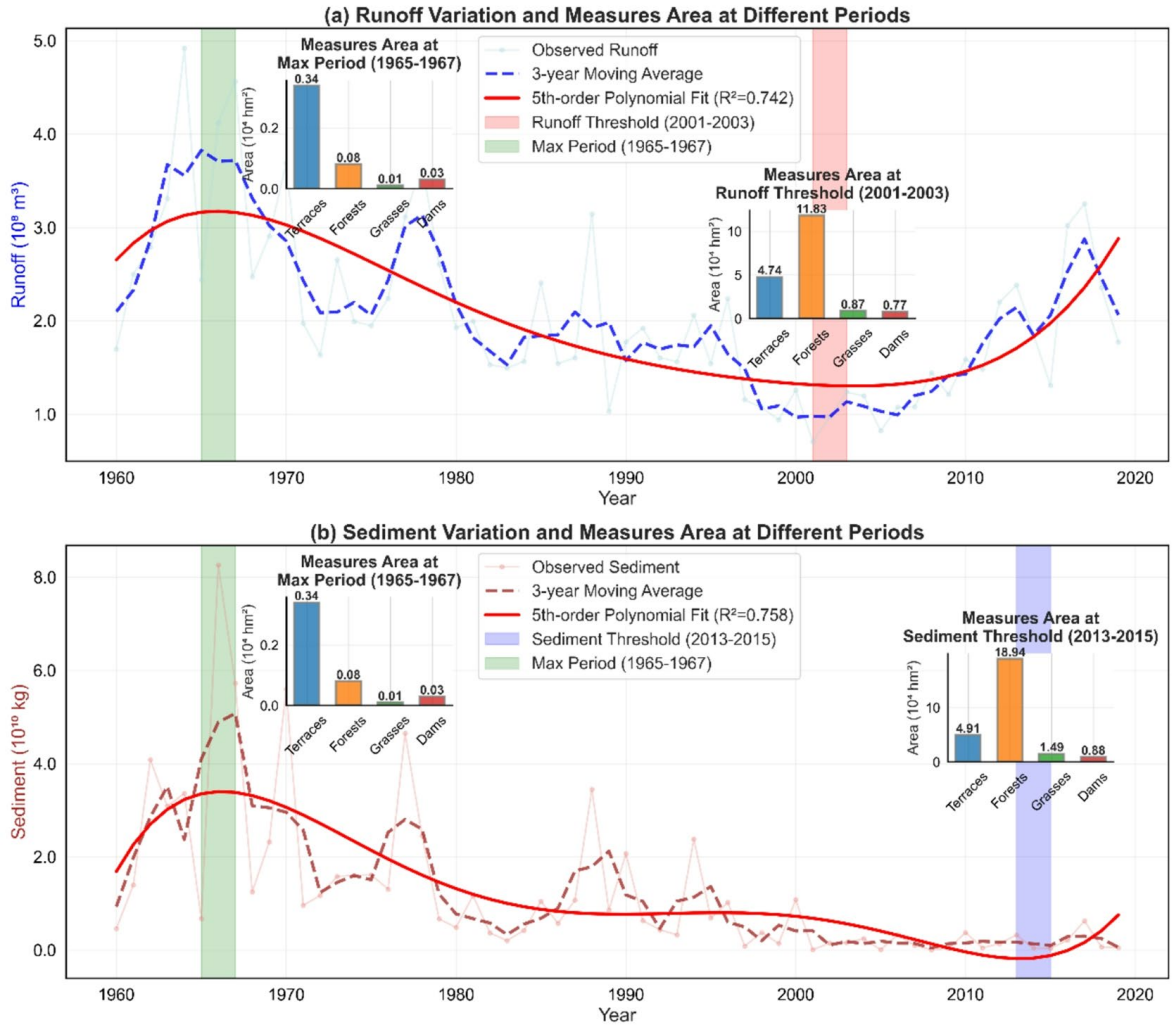
Threshold of Soil and Water Conservation Measures in the Sanchuan River Basin.

Threshold periods refer to specific time windows during which the cumulative implementation area of a conservation measure reaches a critical areal threshold. Once this threshold is surpassed, significant and sustained hydrological benefits can be observed. In this section, we analyze the threshold characteristics of soil and water conservation measures to identify critical periods and associated levels of implementation that significantly enhanced runoff and sediment reduction (Fig. 7). The period 1965–1967 exhibited the highest runoff and sediment loads, reflecting severe soil and water loss. During this time, implementation levels were minimal: terraces covered 0.34 × 10⁴ hm², forests 0.08 × 10⁴ hm², grasses 0.01 × 10⁴ hm², and dams 0.03 × 10⁴ hm² (Fig. 7a). These limited measures were insufficient for effective runoff and sediment control. The period 2001–2003 was identified as the threshold for significant runoff reduction. Average implementation areas increased substantially compared to the 1960s, with terraces reaching 4.74 × 10⁴ hm², forests 11.83 × 10⁴ hm², grasses 0.87 × 10⁴ hm², and dams 0.77 × 10⁴ hm². This expansion, particularly in terraces and forests, drove marked improvements in runoff mitigation. For sediment reduction, the threshold period occurred in 2013–2015, when implementation areas were the highest observed: terraces covered 4.91 × 10⁴ hm², forests 18.94 × 10⁴ hm², grasses 1.49 × 10⁴ hm², and dams 0.88 × 10⁴ hm² (Fig. 7b). The continued increase, especially in forest and grassland areas, was instrumental in achieving peak sediment reduction benefits. The analysis revealed moderate to strong correlations between measure area and corresponding benefits, supported by polynomial fitting results (R² = 0.742 for runoff, 0.758 for sediment). These findings underscore the importance of sustaining and expanding soil and water conservation measures to ensure long-term runoff and sediment reduction. Future strategies should emphasize adaptive management in response to evolving climate and land-use conditions.

Independent validation using non-parametric statistical methods reinforced these thresholds. The Mann-Kendall test confirmed significant decreasing trends in both runoff (Z = − 4.024, *p* < 0.001) and sediment (Z = − 6.333, *p* < 0.001). Pettitt change-point analysis detected 2017 as a statistically significant shift point for runoff (U = 45.0), largely associated with interannual variability following the notably low runoff in 2015 (Fig. 7a). In contrast, the polynomial-identified threshold period of 2001–2003 reflects the inflection in long-term decline, aligning with the cumulative impact of large-scale conservation measures initiated in the early 1990s. For sediment, the Pettitt change point in 2015 falls within the polynomial-derived threshold period of 2013–2015. This agreement suggests that sediment reduction responded to both abrupt change and gradual trend transition as conservation measures reached critical levels of implementation. Together, these results indicate that the identified thresholds represent sustained trend shifts attributable to cumulative conservation effects, whereas methods detecting abrupt mean shifts may capture different hydrological variations. The periods 2001–2003 for runoff reduction and 2013–2015 for sediment reduction thus provide robust markers for when soil and water conservation measures began yielding significant and lasting benefits in the Sanchuan River Basin.

## Discussion

### Seasonal and decadal dynamics of runoff reduction benefits

Our findings reveal distinct seasonal variations in runoff reduction benefits, with peak effectiveness occurring during the summer months (May–September), reaching a maximum of 53.8% in July. This seasonal pattern aligns closely with the region’s concentrated rainfall period and associated soil moisture dynamics. Previous studies have suggested that soil and water conservation (SWC) measures often exhibit enhanced regulatory capacity during extreme hydrological events, a conclusion supported by our results^5,15^. However, the notable decline in runoff reduction benefits during the 2010s (36.0%) compared to the peak in the 2000s (55.5%) warrants closer examination.

Traditionally, such declines have been attributed to increased frequency of extreme rainfall events under climate change^13^. However, our analysis of basin-wide areal rainfall indicates no statistically significant increase in the frequency of daily high-intensity rainfall events (≥ 50 mm) in the 2010s relative to the 2000s. This suggests that climate change may not be the primary driver of the observed decline within this specific period. Instead, the threshold analysis presented in Sect. "Threshold analysis of soil and water conservation measures in the Sanchuan river basin" shows that the areal expansion of key SWC measures—particularly terraces and forests—slowed considerably after reaching their respective benefit threshold periods (2001–2003 for runoff reduction). This plateau in implementation may indicate diminishing marginal returns as measure coverage approaches saturation, or it may reflect a limit to the effectiveness of existing engineering structures under current rainfall regimes.

Furthermore, unquantified factors such as changes in land use patterns, adjustments in farming practices, and modifications to local hydrological connectivity could also modulate runoff responses. Collectively, these observations suggest that sustaining high levels of runoff reduction may require a shift from a strategy focused solely on expanding measure coverage to a more refined adaptive management approach. This could include periodic maintenance and functional upgrades of aging structures, optimized spatial allocation of measures in critical sub-basins, and better integration of SWC planning with overall land-use management to address evolving hydrological pressures^14^. Future research combining distributed hydrological modeling with process-based monitoring would help disentangle the interactions among these various factors.

### Persistence and stability of sediment reduction benefits

In contrast to the fluctuating runoff reduction benefits, sediment reduction benefits remained consistently high throughout the year (> 84% across all months) and exhibited a continuous upward trend from the 1960s to the 2010s, reaching a mean of 97.3% in the latter decade. This result underscores the remarkable long-term success of SWC strategies in controlling soil erosion.

The stability and sustained improvement in sediment reduction can be attributed primarily to the cumulative effects of measures such as afforestation, terrace construction, and improved agricultural practices. These interventions not only directly reduce sediment supply from source areas but also fundamentally diminish sediment transport capacity by altering surface roughness, infiltration rates, and flow pathways^11,21^. Although benefits were slightly lower during the peak flood season of July–August (yet still above 84%), this reflects the persistent challenge of fully controlling sediment transport during extremely high-intensity rainfall events^22,23^. The continuous improvement from the 1960s to the 2010s indicates that the full potential of vegetation recovery, soil structure improvement, and erosion control infrastructure requires considerable time to be realized^12,19^. These findings emphasize the importance of maintaining long-term policy commitment to soil conservation and also suggest that sediment reduction benefits may be more resilient to climatic variability compared to runoff reduction^5,16^.

### Integrated impact of SWC measures and implications for management

The spatiotemporal differentiation of SWC benefits revealed in this study carries important implications for integrated watershed management. The distinct patterns observed in runoff and sediment reduction—in terms of benefit magnitude, seasonal dynamics, and decadal trends—indicate that a single-scale management strategy is unlikely to optimally regulate these two closely related yet distinct hydrological processes simultaneously.

Our results demonstrate that sediment transport processes exhibit stronger lag effects compared to runoff generation (Fig. 4). This reflects the longer “system memory” associated with the complete sequence of sediment detachment, transport, and deposition. The response is influenced not only by contemporaneous rainfall but also profoundly by antecedent hydrological conditions and the existing “stock” of sediment within the watershed^24,25^. Consequently, management practices targeting sediment control require longer lead times and greater consideration of historical hydrological states^26,27^.

The threshold analysis provides a quantitative basis for the scientific planning and resource allocation of SWC measures. The identified critical threshold periods (2001–2003 for runoff reduction and 2013–2015 for sediment reduction) and their corresponding critical implementation areas (e.g., 4.74 × 10⁴ hm² for terraces and 18.94 × 10⁴ hm² for forests) establish clear, phased management targets for the basin. Future SWC strategies should leverage these insights by: (1) implementing anticipatory management based on rainfall forecasts and antecedent soil moisture conditions; (2) maintaining and optimizing the layout and quality of measures around the identified critical area thresholds; and (3) developing adaptive management regimes that account for the differing temporal sensitivities of runoff and sediment responses^28^.

Furthermore, the successful application of Random Forest combined with SHAP analysis in this study demonstrates the strong potential of interpretable machine learning methods for deciphering complex, nonlinear hydrological processes. This approach not only quantifies multi-scale benefits but, more importantly, provides interpretable insights into the contributions of driving factors. It helps transform “black-box” predictions into mechanistically understandable knowledge, offering a powerful new tool for evidence-based decision-making in soil and water conservation.

### Limitations and future directions

While this study, through the integration of long-term data and machine learning, elucidates the multi-scale effects of SWC measures in the Sanchuan River Basin, several limitations should be acknowledged to guide future research.

First, although the Random Forest model performed well in capturing nonlinear relationships, its predictive capability is constrained by the range of the training data, introducing uncertainty when extrapolating to hydrological events beyond historical extremes. Second, the interpolation applied to the SWC measure area data to construct a continuous time series, while necessary, may introduce some uncertainty. Third, and most critically, this study relies primarily on lumped data from the basin outlet control station (Houdacheng Station). While this provides a reliable measure of the integrated basin response, it cannot reveal the spatial heterogeneity of SWC impacts within the watershed. Variations in local topography, soil properties, spatial rainfall distribution, and the spatial configuration of conservation measures may lead to differences in runoff and sediment responses among sub-areas, which are smoothed and masked in the outlet signal. Therefore, the threshold areas identified for terraces and forests (e.g., 4.74 × 10⁴ hm² and 18.94 × 10⁴ hm²) represent the necessary basin-wide totals to achieve the observed benefits, but their optimal spatial configuration remains an open question.

Fourth, the methodological framework adopted in this study for quantifying soil and water conservation (SWC) benefits, which constructs a “without-SWC” baseline scenario based on the 1960s reference period, involves inherent assumptions and uncertainties. This framework attributes long-term deviations from the baseline primarily to the effects of SWC measures. Given the overwhelming scale of SWC implementation in the Loess Plateau region compared to other anthropogenic factors, this constitutes a reasonable and commonly applied approach; however, it inevitably simplifies complex watershed dynamics. Two specific aspects warrant explicit mention: (1) While the operational use of a 50‑mm daily rainfall threshold for identifying high‑erosion events is practical, it may not fully capture the cumulative erosive impact of more frequent, lower‑intensity rainfall events. (2) Other long‑term basin‑scale changes that are not explicitly accounted for in our baseline model—such as gradual shifts in evapotranspiration regimes or adjustments in agricultural water diversions—could also modulate runoff and sediment generation over the six‑decade study period. The absence of a statistically significant increasing trend in high‑intensity rainfall frequency during the critical transition periods (see Sect. "Seasonal and decadal dynamics of runoff reduction benefits") provides supporting evidence for attributing the observed hydrological shifts predominantly to SWC measures. At the same time, the potential influence of the aforementioned unquantified factors indicates that, while robust, the current attribution remains valid within the explicitly stated framework of acknowledged uncertainties.

This limitation does not invalidate the basin-scale conclusions drawn here but clearly indicates that implementation planning requires finer spatial analysis. Future research could incorporate distributed hydrological models or establish nested monitoring networks within the basin to elucidate how the spatial arrangement of measures influences their effectiveness. Such approaches would facilitate the translation of basin-scale aggregate targets into actionable, spatially optimized implementation plans tailored to local conditions.

## Conclusions

This study employed a Random Forest model coupled with SHAP analysis to unravel the multi-scale drivers of runoff and sediment reduction in the Sanchuan River Basin from 1960 to 2019. The principal conclusions are as follows:

1) Runoff reduction is predominantly governed by daily-scale drivers, most notably antecedent rainfall, highlighting the critical role of short-term soil moisture conditions. In contrast, sediment reduction is primarily influenced by annual-scale factors, such as the long-term, cumulative area of conservation measures like afforestation. This fundamental difference underscores that effective management must adopt scale-specific strategies: real-time flood forecasting and management should prioritize recent rainfall patterns, while long-term sediment control requires sustained investment in large-scale ecological engineering.

2) Sediment reduction benefits achieved high levels (~ 97%) and remained consistently high across seasons and decades, demonstrating a robust, cumulative response to long-term conservation efforts. Runoff reduction benefits were not only lower but also exhibited significant seasonal fluctuations and a concerning recent decline. This divergence suggests that the watershed’s response to management is highly process-specific.

3) The identification of distinct threshold periods (2001–2003 for runoff, 2013–2015 for sediment) and their associated critical implementation areas provides concrete, quantitative targets for watershed management planning. These thresholds mark the point at which cumulative SWC efforts begin to yield significant and sustained hydrological benefits, offering a valuable benchmark for other regions.

These findings, derived from the specific context of the Sanchuan River Basin, highlight the necessity of adaptive, multi-scale SWC strategies in the Loess Plateau and provide a methodological framework (RF-SHAP) applicable to other basins facing similar soil erosion and water resource challenges.

## Data Availability

The data supporting this study are available from the corresponding author upon reasonable request.
